# Operational level for unconditional release of contaminated property from affected areas around Fukushima Daiichi nuclear power plant

**DOI:** 10.1093/rpd/nct146

**Published:** 2013-06-17

**Authors:** Haruyuki Ogino, Takatoshi Hattori

**Affiliations:** Radiation Safety Research Center, Nuclear Technology Research Laboratory, Central Research Institute of Electric Power Industry (CRIEPI), 2-11-1 Iwado-kita, Komae, Tokyo 201-8511, Japan

## Abstract

This paper focuses on the surface contamination control of slightly contaminated property after the Fukushima nuclear accident. The operational level for the unconditional release of contaminated properties is calculated in counts per minute (cpm) to enable the use of a typical Geiger-Muller (GM) survey meter with a 50-mm bore, on the basis of the surficial clearance level of 10 Bq cm^−2^ for ^134^Cs and ^137^Cs derived in the previous studies of the authors. By applying a factor for the conversion of the unit surface contamination to the count rate of a survey meter widely used after the Fukushima accident, the operational level for the unconditional release of contaminated properties was calculated to be 2300 cpm on average and 23 000 cpm at the highest-contamination part. The calculated numerical values of the operational levels are effective as long as the typical GM survey meter is used in the radiation measurement.

## INTRODUCTION

Huge amounts of radioactive materials were released into the air during the accident at the Fukushima Daiichi nuclear power plant of Tokyo Electric Power Company triggered by the catastrophic disaster that occurred on 11 March 2011 (e.g. ^131^I: 160 PBq, ^133^Xe: 11 EBq, ^134^Cs: 18 PBq and ^137^Cs: 15 PBq)^(1, 2)^. From the radiation protection viewpoint, health objectives have been to manage and control the emergency and the subsequent existing exposure to ionising radiation so that deterministic effects are prevented and risks of stochastic effects are reduced to an extent reasonably achievable^(3)^. Since the Fukushima accident, emergency responses have been readily taken, such as evacuation, sheltering, temporary relocation, restriction of foodstuffs^(4, 5)^ and decontamination of evacuees^(6)^. A Fukushima health management survey has been conducted^(7)^, and there have been no reports on the observation of deterministic effects even among workers of the nuclear power plant. Note that there are several issues related to stochastic effects due to low-dose and low-dose-rate exposure to ionising radiation from contaminated personal properties (e.g. daily necessities, equipment and vehicles)^(8)^, real properties (e.g. buildings and land) and radioactive wastes (e.g. disaster waste, debris and decontaminated soil)^(9)^. In this study, the authors focus on the surface contamination control of contaminated properties and discuss the operational level for the unconditional release of contaminated properties from affected areas around the Fukushima Daiichi nuclear power plant.

During emergency preparation drills before the Fukushima accident in Japan, the screening level for the surface decontamination of evacuees was set at 13 000 counts per minute (cpm), measured using an ALOKA TGS-136 Geiger-Muller (GM) survey meter with a 50-mm bore, which is one of the most widely used instruments in surface contamination measurement in Japan. The count rate of 13 000 cpm corresponds to a surface contamination density of radioiodine (^131^I) of ∼40 Bq cm^−2^, which is derived from an equivalent dose to the thyroid of 100 mSv for infants. In the derivation of the surface contamination density of ∼40 Bq cm^−2^ for ^131^I, it is assumed that the cumulative radioactive concentration resulting in the above-mentioned equivalent dose to the thyroid in 24 h after the intake is 4×10^−6^ µCi cm^−3^ for infants whose thyroid mass is 4 g, and the surface contamination density is calculated to be 1.4×10^−2^ to 1.4×10^−3^ µCi cm^−2^ using a deposition velocity of 1 to 0.1 cm s^−1^. Thus, the corresponding surface contamination density can be set as 10^−3^ µCi cm^−2^ (∼40 Bq cm^−2^) by using the most conservative deposition velocity (i.e. 0.1 cm s^−1^)^(10)^.

After the nuclear accident, the surrounding situation became so severe, characterised by a high background radiation level, the lack of decontamination tools (e.g. tents, water and tanks) and infrastructure damage due to the earthquake, that the screening level for the surface decontamination of evacuees was relaxed to 100 000 cpm, which is the maximum range of the instrument. To examine the equivalent dose to the thyroid of infants among Fukushima residents, a thyroid monitoring survey was conducted on 26–30 March 2011 for 1080 infants and children (0–15 y old) using TCS-161, TCS-171 and TCS-172 NaI scintillation survey meters. The screening level was set to an ambient dose equivalent rate of 0.2 µSv h^−1^ at an infant's neck^(11)^. This rate corresponds to a residual radioactivity of 4400 Bq of ^131^I, which gives an equivalent dose to the thyroid of 100 mSv for 1-y-old infants if they had chronically inhaled ^131^I for 12 d (from 12 to 23 March). Although the numbers of the examined infants and target areas were limited, no significant signals were detected in 55.4 % of the 1080 infants and children, and the maximum equivalent dose to the thyroid was ∼43 mSv^(12)^.

The same screening level of 100 000 cpm for the evacuees was applied to the surface decontamination of properties removed from the affected areas around the Fukushima Daiichi nuclear power plant for about half a year. On 29 August 2011, the Nuclear Safety Commission (NSC) of Japan gave technical advice to the Nuclear Emergency Response Headquarters (NERH) that the radioactivity of materials on the surface of human skin and properties should be reduced to as low as reasonably achievable, even if the surface contamination levels are lower than the screening level (i.e. 100 000 cpm) to prevent widespread contamination around the affected areas^(13)^. In addition, from the viewpoint of optimising protection against radiation, NSC gave technical advice to progressively change the screening level for decontamination to lower numerical values by taking into account the monitoring results and practical operation of logistical management. Subsequently, on 16 September 2011, NERH ordered the revision of the screening levels from 100 000 to 13 000 cpm for the decontamination of both evacuees and properties^(14)^. The numerical value of 13 000 cpm is equal to the screening level set before the nuclear accident. As of 1 March 2013, the revised screening level of 13 000 cpm has been effective in Japan.

The logic behind the numerical value of 13 000 cpm was originally, as previously noted, the equivalent dose to the thyroid of an infant due to the inhalation of ^131^I. It was numerically calculated from the viewpoint of emergency medical treatment during the nuclear accidents. However, currently, radioiodine (^131^I) is difficult to detect owing to its short half-life (i.e. 8 d), and radiocesiums (^134^Cs and ^137^Cs) have become dominant in the context of radiation dose contributions from various radionuclides. In a technical report on the distribution of radionuclides deposited on the ground after the Fukushima accident^(15)^, the cumulative effective doses in 50 y from the radionuclides ^89^Sr, ^90^Sr, ^110m^Ag, ^129m^Te, ^131^I, ^134^Cs, ^137^Cs, ^238^Pu, ^239^Pu and ^240^Pu were estimated using the dose conversion factors given in IAEA-TECDOC-1162^(16)^, taking account of the external irradiation and inhalation of resuspended radionuclides. The result shows that 96.4 % of the total effective dose is given by ^137^Cs, 3.4 % by ^134^Cs and 0.15 % by ^110m^Ag. Regarding other radiological countermeasures taken after the accident, contaminated foodstuffs had been restricted according to provisional regulation values for both radioiodines and radiocesiums in an initial stage after the accident. Note that revised food safety regulation limits have been implemented since 1 April 2012, which provide the concentrations of only radiocesiums^(5)^. Disaster waste and debris have also been managed by considering the concentration of radiocesiums. Hereby, it is appropriate to similarly control and manage the unconditional release of properties in the existing exposure situation following the Fukushima nuclear accident in consideration of the radiocesium levels of contamination. In this study, the operational levels for the unconditional release of contaminated properties from the affected areas in units of counts per minute (cpm) measured using typical GM survey meters are derived by assuming radiocesium contamination, and the practical implementation is discussed.

## DOSE CRITERIA FOR UNCONDITIONAL RELEASE OF PROPERTY

When contaminated properties are released from affected areas around the Fukushima Daiichi nuclear power plant, followed by an appropriate surface contamination measurement at a certain operational level, to similarly affected areas or unaffected areas, the external and internal doses of radiation caused by handling the contaminated properties and the associated radiation risk become the main concern. The trade of contaminated commodities and consumer products manufactured using contaminated materials outside the contaminated area should be in accordance with the rules or recommendations for international trade^(17)^. A system of radiation protection measures recommended by the International Commission on Radiological Protection (ICRP) applies to all radiation exposure from any controllable sources, and contaminated properties should be considered in such a manner. If properties are highly contaminated, then their surface should be decontaminated following specified procedures to prevent deterministic effects and manage the possibility of stochastic effects. However, if the surface contamination levels are low enough, a need for exemption levels for decontamination to avoid excessive regulatory procedures can be argued. In this respect, there is an important description in the ICRP recommendations^(3)^ that a graded burden of obligation must be foreseen according to the amenability of a particular source to regulatory control and the level of associated risk. This graded approach leads to a fundamental concept that delineates the extent of radiation protection control, namely, an ‘exemption’ from some or all radiation protection regulatory requirements. A basic consideration behind the concept is that such controls are often regarded as unwarranted in a situation where it is perceived that the effort to impose control is excessive compared with the trivial associated individual risks^(3)^.

Regarding the derivation of trivial individual risks or doses, ICRP has developed a radiation-risk-based approach and a natural-radiation-based approach^(17)^. First, there has been an internationally held view that few people would commit their own resources to reduce an annual risk of death of 10^−5^, and even fewer would take action at an annual risk of death of 10^−6^. Thus, ICRP set the annual risk of death that is considered of no concern to an individual at 10^−6^ to 10^−7^, and the trivial individual effective dose was derived to be on the order of magnitude of 10–100 µSv y^−1^ using a nominal risk coefficient of ∼5×10^−2^ Sv^−1^ for whole-body exposure as a broad average over age and sex. Second, natural radiation sources give an average individual dose of ∼2.4 mSv y^−1^ as a world average^(18)^, which conceals a wide variation due to differences between concentrations of radioactive materials in the ground and in building materials, as well as to differences in altitude and lifestyle. Individual members of the public do not generally take account of the variation in exposure to natural radiation sources when considering moving from one part of the country to another, although about half of the natural radiation dose originates from exposure to cosmic radiation, terrestrial rays and radionuclides in the body, for which control is impractical. Therefore, it was decided that a dose that is small in comparison with the variation can be regarded as trivial. On this basis, an effective dose on the order of 1–5 % of the natural background (i.e. 20–100 µSv y^−1^) was suggested as the dose criterion for exemption.

The important part of the above-described argument is that the dose criterion of a trivial individual dose of tens of µSv y^−1^ was set in consideration of the annual risk of death and the variation in the natural radiation level and that trivial individual doses are in the effective dose band of 10–100 µSv y^−1^. Regarding the set range of one order of magnitude, the fixed numerical value of 10 µSv y^−1^ (i.e. the lower level of the band) has often been used to derive relevant regulatory values such as exemption and unconditional clearance levels in units of radioactive volumetric concentration (Bq g^−1^) and surficial density (Bq cm^−2^). It should be noted that the considerations in the derivation of trivial individual dose bands leave a safety margin in the practical judgment of the unconditional release of properties from the viewpoint of surface contamination measurement using the operational levels discussed in this study.

## DERIVATION OF OPERATIONAL LEVELS

### Surficial clearance level

On the basis of the fundamental concepts of exemption and trivial individual doses, the authors have derived surficial clearance levels in units of Bq cm^−2^ in the previous studies of the authors for representative radionuclides including radiocesiums by developing an original methodology of assessing doses resulting from handling surface-contaminated objects^(19–21)^. In the method developed, surface-contaminated objects were classified into three general categories, namely, manually handled objects, closely handled objects and remotely handled objects, and annual individual effective doses from external exposure, committed effective doses from internal exposure (i.e. ingestion and inhalation) and the annual equivalent dose of the skin due to the deposition of radioactive materials on the human body were calculated. Note that one is not concerned here with the assessment of exposure from enriched radioactivity due to the combustion of objects. Therefore, it can be considered that the operational levels determined in this study are not applicable to flammable properties. Table 1 shows the results of the authors’ calculation of surficial clearance levels in units of Bq cm^−2^ for representative radionuclides generated at a typical nuclear power plant, which correspond to exemption dose criteria of 10 µSv y^−1^ for realistic dose assessment and 1 mSv y^−1^ for low-probabilistic dose estimation.

**Table 1.** Calculation of surficial clearance levels in units of Bq cm^−2^ in the author's previous studies ^(19–21)^.

Category	Manually handled objects		Closely handled objects		Remotely handled objects		Critical pathway		Surficial clearance levels (Rounded value, Bq/cm^2^)
Pathway	Ingestion	Skin	External	Inhalation	External	Inhalation			
Nuclide/no.	1	2	3	4	5	6			
^3^H	7.2×10^3^	N.A	N.A	2.8×10^2^	N.A	1.9×10^3^	4	2.8×10^2^	100
^14^C	5.2×10^2^	6.4×10^2^	N.A	1.1×10^4^	N.A	7.7×10^4^	1	5.2×10^2^	1000
^36^Cl	1.3×10^2^	2.2×10^2^	5.7×10^4^	2.7×10^3^	1.2×10^5^	1.8×10^4^	1	1.3×10^2^	100
^41^Ca	1.6×10^3^	N.A	1.2×10^4^	7.3×10^4^	2.4×10^12^	4.9×10^5^	1	1.6×10^3^	1000
^54^Mn	4.2×10^2^	9.1×10^3^	1.5×10^1^	1.2×10^4^	3.4×10^1^	8.2×10^4^	3	1.5×10^1^	10
^55^Fe	4.0×10^2^	5.4×10^4^	6.0×10^10^	4.9×10^4^	1.3×10^11^	3.2×10^5^	1	4.0×10^2^	1000
^60^Co	3.3×10^1^	2.6×10^3^	4.0×10^0^	2.1×10^3^	8.5×10^0^	1.4×10^4^	3	4.0×10^0^	10
^59^Ni	2.5×10^3^	9.4×10^3^	5.0×10^5^	1.2×10^5^	1.1×10^6^	8.1×10^5^	1	2.5×10^3^	1000
^63^Ni	1.0×10^3^	3.0×10^4^	N.A	4.0×10^4^	N.A	2.7×10^5^	1	1.0×10^3^	1000
^65^Zn	8.2×10^1^	6.4×10^3^	2.7×10^1^	8.7×10^3^	5.8×10^1^	5.8×10^4^	3	2.7×10^1^	10
^90^Sr	1.2×10^1^	1.9×10^2^	4.1×10^6^	1.8×10^2^	9.4×10^6^	1.2×10^3^	1	1.2×10^1^	10
^94^Nb	8.6×10^1^	2.5×10^2^	5.4×10^0^	1.9×10^3^	1.2×10^1^	1.3×10^4^	3	5.4×10^0^	10
^99^Tc	1.7×10^2^	3.5×10^2^	N.A	4.3×10^3^	N.A	2.9×10^4^	1	1.7×10^2^	100
^129^I	3.8×10^0^	8.4×10^2^	1.8×10^2^	2.7×10^2^	3.7×10^2^	1.8×10^3^	1	3.8×10^0^	10
^134^Cs	5.3×10^1^	2.9×10^2^	6.5×10^0^	1.8×10^3^	1.4×10^1^	1.2×10^4^	3	6.5×10^0^	10
^137^Cs	6.5×10^1^	2.6×10^2^	1.5×10^1^	2.1×10^3^	3.1×10^1^	1.4×10^4^	3	1.5×10^1^	10
^152^Eu	1.2×10^2^	3.4×10^2^	7.7×10^0^	5.3×10^2^	1.6×10^1^	3.5×10^3^	3	7.7×10^0^	10
^154^Eu	7.3×10^1^	1.6×10^2^	8.2×10^0^	4.2×10^2^	1.7×10^1^	2.8×10^3^	3	8.2×10^0^	10
^239^Pu	2.0×10^0^	3.9×10^5^	6.0×10^3^	4.3×10^−1^	1.4×10^4^	2.9×10^0^	4	4.3×10^−1^	1
^241^Am	2.3×10^0^	8.0×10^3^	1.8×10^2^	5.1×10^−1^	3.8×10^2^	3.4×10^0^	4	5.1×10^−1^	1

The calculation code QAD-CGGP2^(22)^ was used to calculate the effective dose of external irradiation. The committed effective dose of inhalation was assessed using the resuspension rate (h^−1^), contaminated surface area (m^2^), room volume (m^3^), exchange rate (h^−1^), breathing rate (m^3^ h^−1^) and the dose coefficient of inhalation for each radionuclide (Sv Bq^−1^). The committed effective dose of ingestion was assessed using the transfer factor from a contaminated surface to the hands, the transfer factor from the hands to the mouth, the frequency of ingestion (h^−1^) and the dose coefficient of ingestion for each radionuclide (Sv Bq^−1^). The dose coefficients of inhalation and ingestion are given in ICRP Publication 68^(23)^ for workers and in Publication 72^(24)^ for members of the public. These methodologies to derive surficial clearance levels are consistent with the recently developed stylised approaches applied in the transport safety field in IAEA (e.g. given in TECDOC-1149^(25)^) and in the radiation and waste safety fields (e.g. given in Safety Guide No. RS-G-1.7^(26)^). Details of the parameters used for calculating the volumetric clearance levels given in the Safety Guide No. RS-G-1.7 are further provided in the Safety Report Series No. 44^(27)^ and are also incorporated into the methodology the authors developed for surface contamination, such as the annual exposure time and decay time before or during the assumed exposure scenario. Recently, IAEA has published revised International Basic Safety Standards (BSS) entitled General Safety Requirements Part 3^(28)^ in the latest hierarchy of Safety Standards. This provides the exemption levels for moderate amounts of materials in units of Bq as a total radioactivity and in Bq g^−1^ as a radioactive volumetric concentration, which is given in the BSS 1996 edition^(29)^, and the exemption levels for bulk amounts of solid materials and clearance levels for solid materials in units of Bq g^−1^, which are given in Safety Guide No. RS-G-1.7^(26)^.

For the radiocesiums, the surficial clearance levels were calculated to be 10 Bq cm^−2^ for ^134^Cs and ^137^Cs as rounded numerical values, as shown in Table 1. Before the volumetric clearance levels given in the Safety Guide^(26)^ were finally introduced into the relevant national laws for the first Japanese decommissioning of commercial nuclear power plants^(30)^, NSC calculated their own volumetric clearance levels and compared them with international clearance levels^(31)^. In the calculation, surficial clearance levels were also given, although they were not introduced into the laws, by assessing the effective doses from external irradiation, inhalation and ingestion, which arise from reusing surface-contaminated metal objects such as pumps, motors and valves. The surficial clearance levels for ^134^Cs and ^137^Cs were calculated to be 12 and 33 Bq cm^−2^, respectively^(31)^. Although the assessments are limited to the re-use of metals and thus do not represent all assumed exposure scenarios for the unconditional release of contaminated properties, it can be considered that the results partly validate the derived surficial clearance levels of 10 Bq cm^−2^ for ^134^Cs and ^137^Cs. Further details of the methodology of deriving the surficial clearance levels given in Table 1 have been provided in the previous papers of the authors^(19–21)^.

### Measurement and evaluation of surface contamination

In the measurement and evaluation of surface contamination by a direct measurement of radioactivity, surface contamination density is given by equation (1), in accordance with the Japanese Industrial Standards Committee (JIS4504)^(32)^ and International Organization for Standardization (ISO7503)^(33)^, where *A_i_* (Bq cm^−2^) is the surface density of radioactive contamination for radionuclide *i*, *N* (counts per second, cps) is the measured count rate, *N_b_* (cps) is the background count rate, *ɛ_e,i_* is the instrument efficiency for radionuclide *i*, *W* (cm^2^) is the effective window area of the detector and *ɛ_s,i_* is the source efficiency for radionuclide *i*. Note that the surface contamination area is greater than or equal to the effective window area of the detector and that the surface contamination density within the effective window area of the detector is uniform^(32)^.

The operational level for the unconditional release of contaminated properties discussed in this study corresponds to the measured count rate (*N*) in equation (1). The background radiation count rate (*N_b_*) is considered to be zero in the calculation of the operational level considering conservatism. The effective window area is ∼20 cm^2^ in the case of a 50-mm bore. The source efficiency of properties contaminated with radiocesiums (^134^Cs and ^137^Cs) is set at 0.5 in accordance with JIS4504^(32)^ and ISO7503^(33)^, unless otherwise specified, considering that the maximum beta-ray energies of the radiocesiums are higher than 0.4 MeV (i.e. 0.658 MeV for ^134^Cs and 0.514 MeV for ^137^Cs), although an experimental study in Japan has shown a higher source efficiency for various materials^(34, 35)^. Instrument efficiency (*ɛ_e,i_*) is dependent on the type of GM survey meter used. Counting efficiency is defined as the product of the source efficiency (*ɛ_s,i_*) and the instrument efficiency (*ɛ_e,i_*):
(1)
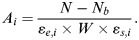



In the screening examination for the decontamination of the evacuees and unconditionally released properties from the areas affected by the Fukushima nuclear accident, a TGS-146B GM survey meter with a 50-mm bore, as well as a TGS-136 GM survey meter, has been widely used, as previously noted^(1, 2)^. With regard to the relationship between count rate [kilocounts per minute (kcpm)] and surface contamination density (Bq cm^−2^), the Japan Nuclear Energy Safety Organization (JNES), an incorporated administrative agency in Japan, evaluated the conversion factors (Bq cm^−2^ kcpm^−1^) on the basis of calibration experiments using the TGS-146B GM survey meter. In one of its reports, JNES discusses the external exposure doses of car mechanics during the maintenance of cars from the restricted area^(36)^. From the discussion, Table 2 shows a summary of the basic performance of a typically used GM survey meter after the Fukushima accident (i.e. TGS-146B) for representative sources including radiocesiums (i.e. ^36^Cl, ^60^Co, ^134^Cs, ^137^Cs and U_3_O_8_). The counting efficiency for each source is here calculated using the conversion factors provided by the JNES report^(36)^.

**Table 2.** Conversion factor and counting efficiency of typically used GM survey meter in Fukushima.

Source	Maximum beta-ray energy [MeV]	Conversion factor [Bq cm^−2^ kcpm^−1^]	Counting efficiency
^36^Cl	0.709	3.55	0.240
^60^Co	0.318	6.82	0.125
^134^Cs	0.658	4.16	0.204
^137^Cs	0.514	4.36	0.195
U_3_O_8_	2.29	2.66	0.320
^134+137^Cs^a^	—	4.28	0.199

^a^The radioactivity ratio of ^134^Cs/^137^Cs is 0.64 as of February 2013.

### Operational levels for unconditional release of contaminated properties

The operational levels for the unconditional release of contaminated properties (cpm) were derived by dividing the surficial clearance levels (Bq cm^−2^) given in Table 1 by the conversion factors (Bq cm^−2^ kcpm^−1^) given in Table 2. For materials containing a mixture of radionuclides, exemption or clearance can generally be applied depending on whether the following equation is satisfied:
(2)
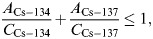

where *A_Cs-134_* and *A_Cs-137_* represent actual measurement results of surface contamination density (Bq cm^−2^) for ^134^Cs and ^137^Cs, respectively, and *C_Cs-134_* and *C_Cs-137_* represent surficial clearance levels (Bq cm^−2^).

By applying the surficial clearance level of 10 Bq cm^−2^ for both ^134^Cs and ^137^Cs given in Table 1, the following equation is obtained:
(3)
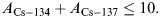



**Figure 1.**
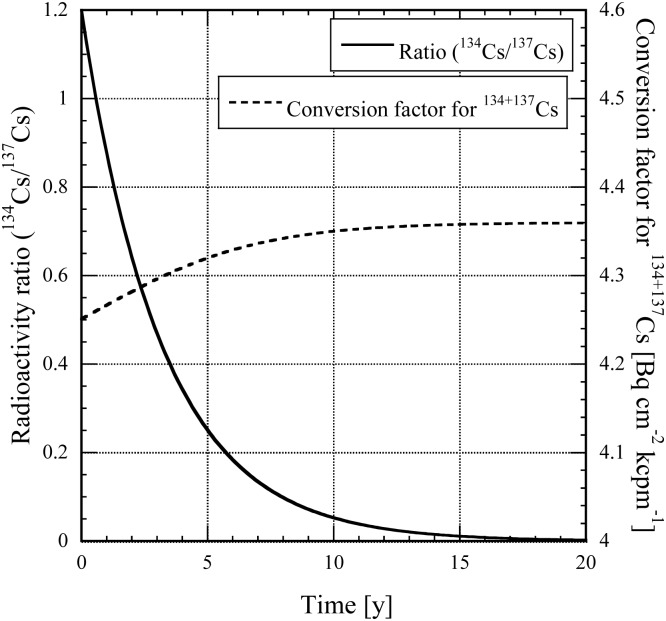
Radioactivity ratio and conversion factor for ^134^Cs and ^137^Cs as functions of time.

The radioactivity ratio of ^134^Cs to ^137^Cs was estimated to be 1.2 in the initial phase of the Fukushima accident by the Nuclear Safety Agency of Japan, which shows that 18 PBq of ^134^Cs and 15 PBq of ^137^Cs were released into the air from reactor units 1–3 of the Fukushima Daiichi nuclear power plant during the period of 11–16 March 2011^(1, 2)^. Owing to the difference in half-life (i.e. 2.1 y for ^134^Cs and 30 y for ^137^Cs), the radioactivity ratio of ^134^Cs to ^137^Cs has decreased to 0.64 as of February 2013 and the conversion factor has reached 4.28 Bq cm^−2^ kcpm^−1^, which was calculated by weighting the conversion factors for ^134^Cs and ^137^Cs by the radioactivity ratio (i.e. (4.16 × 0.64 + 4.36)/(1 + 0.64) = 4.28). With time, the ratio will decrease and the conversion factors (Bq cm^−2^ kcpm^−1^) will saturate to become those for ^137^Cs as shown in Figure 1. By dividing equation (3) by the conversion factor of 4.28 Bq cm^−2^ kcpm^−1^ and by the saturated conversion factor of 4.36 Bq cm^−2^ kcpm^−1^ for ^137^Cs, an operational level for the unconditional release of contaminated properties is obtained from the affected area of 2300 cpm for the TGS-146B GM survey meter, which is one of the typical instruments used in the affected area around the power plant and also used by NSC in deriving the screening level after the Fukushima accident.
(4)
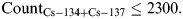

## DISCUSSION

In considering the practical implementation of the above-calculated operational level of 2300 cpm for the daily routine for the unconditional release of contaminated properties from affected areas around the Fukushima Daiichi nuclear power plant, there are a number of factors that can cause confusion in the practical field and disturb efficient operation, such as the dependence on measurement instruments with different counting efficiencies, the effect of the surface condition of the properties with different source efficiencies and the distribution of surface contamination (whether to judge by the average or highest level). Here, on the basis of these observations after the Fukushima nuclear accident, the authors propose the operational levels for the unconditional release of contaminated property derived in this study and important points taken from the viewpoints of radiation measurement and radiation protection to ensure the efficient implementation of the operational level to the practical situation in Japan as follows.

### Points to note from the viewpoint of radiation measurement

The above-calculated operational level of 2300 cpm is considered to be effective as long as the TGS-146B GM survey meter is used in the surface contamination measurement, because the conversion factor (Bq cm^−2^ kcpm^−1^) is dependent on the counting efficiency of the radiation measurement instrument used. In the case of instruments with the same bore size (i.e. 50 mm), for instance, a TCS-319H scintillation detector for beta-ray measurement manufactured by Aloka has an instrument efficiency of 45.8 % for ^137^Cs; thus, the operational level can be calculated as 2700 cpm using equation (1) and assuming that the source efficiency is 0.5 in accordance with JIS4504^(32)^ and ISO7503^(33)^. An SPS-206-F1 plastic scintillation detector for beta-ray measurement manufactured by Ohyo Koken Kogyo Co., Ltd., has a conversion factor of 4.72 Bq cm^−2^ kcpm^−1^ for ^137^Cs; thus, the operational level can be calculated as 2100 cpm by similarly dividing equation (3) by its conversion factor. Furthermore, an SPS-206Z plastic scintillation detector for beta-ray measurement, also manufactured by Ohyo Koken Kogyo Co., Ltd., has a relatively high conversion factor of 6.07 Bq cm^−2^ kcpm^−1^ for ^137^Cs; thus, the operational level can be calculated as 1700 cpm.

In addition to the dependence on the type of radiation measurement instrument used, the effect of source efficiency for the materials of the contaminated properties should also be taken into account when the operational level for unconditional release is set. Past experimental studies on the source efficiency of representative radionuclides for various materials^(37, 38)^ show that the source efficiency of 0.5 for ^137^Cs is effective as long as the deposited radioactive substances are located near the surface of impermeable materials, such as metals (e.g. aluminium, iron, lead and stainless steel), glass, acrylic, vinyl chloride, rubber and wood^(34, 35)^. In the case of permeable materials, on the other hand, the source efficiency becomes lower than 0.5 owing to the self-shielding effect (e.g. 0.3 for concrete coated with water paint in the case of ^137^Cs^(34, 35)^), even if the maximum beta-ray energy is higher than 0.4 MeV as shown in Table 2. Considering that the operational levels calculated using equation (1) are inversely proportional to the source efficiency, the source efficiency should be set to avoid the underestimation of radioactivity on the surface of released properties.

Taken together, it is noteworthy that the appropriate operational level for the unconditional release of contaminated properties should be preliminarily defined for each radiation measurement instrument used and the material of the contaminated property released, considering the dependence of the operational level on the counting efficiency and source efficiency.

### Points to note from the viewpoint of radiological protection

In the application of the screening level of 13 000 cpm for the release of contaminated properties after the Fukushima nuclear accident in Japan, it has been required that the highest level among the whole surface contamination on the properties should be lower than the screening level. However, regarding the proposed requirement of surface contamination measurement to decide the unconditional release of properties discussed in this study, it is important to remember that the fixed numerical value of 10 µSv y^−1^ has been rigidly used to determine the surficial clearance levels (i.e. 10 Bq cm^−2^ for ^134^Cs and ^137^Cs), while the dose criteria of a trivial individual dose of tens of µSv y^−1^ were developed considering the annual risk of death and the variation in the range of the natural radiation level of one order of magnitude (i.e. 10–100 µSv y^−1^). In this respect, NSC suggested in its report on clearance levels for nuclear facilities published in 2004^(30)^ that the average concentration of radioactivity should be made lower than the volumetric clearance level (e.g. 100 Bq kg^−1^ for ^137^Cs) and that the highest allowable concentration of radioactivity also should be made lower than tenfold the volumetric clearance level at the same time (e.g. 100×10=1000 Bq kg^−1^), from the viewpoint of avoiding excessive regulatory procedures. It is also appropriate to apply the same approach to surface contamination measurement and the decision for the unconditional release of contaminated properties to rationally control non-uniformly distributed radionuclides. Namely, the average level of radioactivity contamination on the surface should be below the operational level for surficial clearance (i.e. 2300 cpm for ^134^Cs and ^137^Cs) and the highest level of radioactivity contamination should also be below tenfold the operational level for surficial clearance (i.e. 23 000 cpm).

Finally, in this study, the authors determine the operational level for the unconditional release of contaminated properties from affected areas around the Fukushima Daiichi nuclear power plant to unaffected or minimally affected areas, considering the international rules of exemption and clearance, which is based on an additional trivial individual dose band (i.e. 10 to 100 µSv y^−1^). However, given the notion that contaminated properties are conditionally released within similarly affected areas, protective actions should be planned and optimised using the concept of reference levels for existing controllable exposure situations recommended in the ICRP 2007 Recommendations^(3)^. As a long-term objective, the reference level for the optimisation of protection of people living in the affected areas should be selected from the lower part of the 1–20 mSv y^−1^ range, and past experience has shown that the typical reference level is 1 mSv y^−1(39)^, which is also given as the intervention exemption level for public protection in prolonged-exposure situations^(40)^. Considering this recommendation, it can be considered that the conditional release of contaminated materials within similarly affected areas should be set using the dose of around 1 mSv y^−1^ as the intervention exemption level, and a higher operational level for the conditional release can be derived in such a case.

## CONCLUSION

The operational level for the unconditional release of contaminated properties from affected areas around the Fukushima Daiichi nuclear power plant was calculated. The results indicated that the average level of radioactivity contamination on the surface should be below 2300 cpm and that the highest level of radioactivity contamination should be below 23 000 cpm when using a typical GM survey meter (TGS-146B) for measurement. It is important to preliminarily define the appropriate operational level by taking into account the counting efficiency of the radiation measurement instrument used and the source efficiency of the contaminated properties released.

## FUNDING

This work was supported by the Radiation Safety Research Project of the Central Research Institute of Electric Power Industry (CRIEPI). Funding to pay the Open Access publication charges for this article was provided by CRIEPI.
